# Soybean Viromes in the Republic of Korea Revealed by RT-PCR and Next-Generation Sequencing

**DOI:** 10.3390/microorganisms8111777

**Published:** 2020-11-12

**Authors:** Yeonhwa Jo, Young Nam Yoon, Yun-Woo Jang, Hoseong Choi, Yeong-Hoon Lee, Sang-Min Kim, Soo Yeon Choi, Bong Choon Lee, Won Kyong Cho

**Affiliations:** 1 Research Institute of Agriculture and Life Sciences, College of Agriculture and Life Sciences, Seoul National University, Seoul 08826, Korea; yeonhwajo@gmail.com (Y.J.); bioplanths@gmail.com (H.C.); 2 Crop Production Technology Research Division, National Institute of Crop Science, Rural Development Administration, Miryang 50424, Korea; yoonyn@korea.kr (Y.N.Y.); ywj2012@korea.kr (Y.-W.J.); 3 Bioenergy Crop Research Institute, National Institute of Crop Science, Rural Development Administration, Muan 58545, Korea; sky3832@korea.kr; 4 Crop Foundation Division, National Institute of Crop Science, Rural Development Administration, Wanju 55365, Korea; kimsangmin@korea.kr (S.-M.K.); choisy99@korea.kr (S.Y.C.)

**Keywords:** Korea, mutation, RNA-seq, soybean, viral genome, virome, virus

## Abstract

Soybean (*Glycine max* L.) is one of the most important crop plants in the Republic of Korea. Here, we conducted a soybean virome study. We harvested a total of 172 soybean leaf samples showing disease symptoms from major soybean-growing regions in the Republic of Korea. Individual samples were examined for virus infection by RT-PCR. Moreover, we generated eight libraries representing eight provinces by pooling samples and four libraries from single samples. RNA-seq followed by bioinformatics analyses revealed 10 different RNA viruses infecting soybean. The proportion of viral reads in each transcriptome ranged from 0.2 to 31.7%. Coinfection of different viruses in soybean plants was very common. There was a single dominant virus in each province, and this geographical difference might be related to the soybean seeds that transmit viruses. In this study, 32 viral genome sequences were assembled and successfully used to analyze the phylogenetic relationships and quasispecies nature of the identified RNA viruses. Moreover, RT-PCR with newly developed primers confirmed infection of the identified viruses in each library. Taken together, our soybean virome study provides a comprehensive overview of viruses infecting soybean in eight geographical regions in the Republic of Korea and four single soybean plants in detail.

## 1. Introduction

Soybean (*Glycine max* L. (Merrill)), a legume species native to East Asia, is widely cultivated and consumed in many Asian countries. Fermented soybeans are used to make soy sauce, soybean paste, and tempeh, while unfermented soybeans are used to make soymilk and tofu. In addition, soybeans provide soybean oil, which is important for both domestic and industrial purposes.

Diverse diseases and insects can seriously affect soybean production [1]. Of the known pathogens, several viruses infecting soybean are major problems [2]. *Soybean mosaic virus* (SMV) in the genus *Potyvirus* (the family *Potyviridae*) is the most prevalent and destructive soybean virus in the world [2]. In the United States, seven different strains (G1–G7) from various SMV isolates have been classified according to their response to susceptible and resistant soybean cultivars [3], whereas 21 different SMV strains (SC1–SC21) have been identified in China [4]. Moreover, *Bean pod mottle virus* (BPMV) in the genus *Comovirus* (the family *Secoviridae*) has been widespread in the United States, reducing yield and seed quality [5].

In the Republic of Korea, soybean is one of the most important crops [6], and it is usually planted in May and harvested in October. Several viruses infecting soybean have been identified in the Republic of Korea [7,8]. Of the known viruses infecting soybean, two novel viruses—*Soybean yellow mottle mosaic virus* (SYMMV) in the genus *Carmovirus* [9] and *Soybean yellow common mosaic virus* (SYCMV) in the genus *Sobemovirus* [10]—have been identified in Korea. Based on previous reports, four viruses—*Peanut stunt virus* (PSV) in the genus *Cucumovirus*, SMV, SYMMV, and SYCMV—have been frequently identified in Korea [7,8]. Moreover, infection of *Peanut mottle virus* (PeMoV), which is a member of the genus *Potyvirus* in the family *Potyviridae*, infecting soybean in the Republic of Korea has been previously reported [11].

The development of next-generation sequencing (NGS) facilitates the identification and diagnosis of plant viruses, including novel viruses [12,13,14]. In addition, NGS has been widely used for several plant virome studies [15,16,17,18]. However, few studies have identified viruses infecting soybean using NGS [19].

Here, we carried out a comprehensive soybean virome study in the Republic of Korea using reverse-transcription-polymerase chain reaction (RT-PCR) and RNA sequencing (RNA-seq). We collected a total of 172 soybean leaf samples showing disease symptoms from major soybean-growing regions in the Republic of Korea. We examined infection of virus in 172 soybean leaf samples by RT-PCR. In addition, we sequenced eight different libraries from eight different geographical regions and four libraries from four single soybean plants showing viral disease symptoms. Bioinformatics analyses and RT-PCR revealed the soybean viromes in the Republic of Korea in detail.

## 2. Materials and Methods

### 2.1. Collection of Soybean Leaf Samples

Soybean leaf samples displaying viral disease symptoms, including mosaic, mottling, chlorosis, stunting, and yellowing, were collected in June 2016 from major soybean-growing regions in the Republic of Korea. The collected leaf samples were immediately frozen in liquid nitrogen. Frozen individual leaf samples were ground with a plastic grinding pestle. An individual leaf sample was used for total RNA extraction followed by RT-PCR. For NGS, the same amount of fine powder from each leaf sample was pooled according to the eight different provinces. Based on RT-PCR results, we selected four single leaf samples that were severely coinfected by different viruses for NGS.

### 2.2. Total RNA Extraction, Library Preparation, and NGS

Each individual leaf sample was ground in a mortar with a pestle and used for total RNA extraction using the RNeasy Plant Mini Kit according to the manufacturer’s instructions (Qiagen, Hilden, Germany). We isolated mRNA from the extracted total RNA using the NEBNext Poly(A) mRNA Magnetic Isolation Module according to the manufacturer’s instructions (NEB, Ipswich, MA, USA). We generated the RNA-seq libraries using the NEBNext Ultra™ RNA Library Prep Kit for Illumina (NEB) according to the manufacturer’s instructions. Each library was indexed using NEBNext Multiplex Oligos for Illumina (Index Primers Set 1) (NEB). The 12 libraries were paired-end (100 bp X 2) sequenced by Illumina’s HiSeq 2000 system (Macrogen, Seoul, Korea). All raw sequence data as fastq files from the 12 libraries were deposited in the National Center for Biotechnology Information (NCBI)’s sequence read archive (SRA) database with respective accession numbers under BioProject accession number PRJNA637168.

### 2.3. De Novo Transcriptome Assembly and BLASTX Search

The obtained raw sequence reads were de novo assembled by the Trinity program with default parameters as described previously [20]. The obtained contigs were subjected to BLASTX search with E-value 1e-10 as a cutoff against the plant viral database derived from the NCBI (https://www.ncbi.nlm.nih.gov/genome/viruses/). In order to eliminate non-viral sequences, the obtained virus-associated contigs were again subjected to BLASTX search against the NCBI non-redundant (NR) protein database. As a result, we obtained only virus-associated contigs from each library.

### 2.4. Genome Assembly and Annotation for Identified Viruses

In order to assemble complete or nearly complete genomes of identified viruses, each virus-associated contig was aligned to the reference viral genomes using the ClustalW program implemented in the MEGA7 program [21]. Some virus-associated contigs covered nearly complete viral genome sequences. We deleted poly(A) tail sequences at the 3′ terminal. In addition, raw sequence reads were aligned to the reference genome to check the sequences of assembled viral genomes using a Burrows–Wheeler Aligner (BWA) program with default parameters [22]. The ORFfinder program (https://www.ncbi.nlm.nih.gov/orffinder/) was used to predict open reading frames (ORFs) in each virus genome. All viral genome sequences covering whole ORFs were deposited in the NCBI GenBank database with respective accession numbers.

### 2.5. Construction of Phylogenetic Trees for Identified Viruses

For the phylogenetic tree construction, we retrieved viral genome sequences covering at least complete ORFs from NCBI’s GenBank for each identified virus species by BLASTN search. Assembled viral genome sequences and the retrieved genome sequences for each virus species were aligned by the MAFFT program with the L-INS-I option [23]. Aligned nucleotide sequences were trimmed by the trimAL program with the automated 1 option [24]. Trimmed nucleotide sequences were subjected to ModelFinder implemented in the IQ-TREE program to select the best-fit model according to the Bayesian information criterion (BIC) [25]. The best-fit models for each virus species were TN+F+I (PeMoV), TIM2+F+I+G4 (PSV_RNA1), TN+F+G4 (PSV_RNA2), TIM2+F+G4 (PSV_RNA3), GTR+F+I+G4 (SMV), TIM2e+I+G4 (SYMMV), TIM2e+I+G4 (SYCMV), and GTR+F+I+G4 (WVMV). The phylogenetic trees for each virus species were inferred by IQ-TREE using the selected best-fit model, Ultrafast Bootstrap with 1000 iterations [26,27], and the SH-aLRT branch test [28]. The generated phylogenetic trees for individual virus species were visualized using the FigTree (version 1.4.4) program (https://github.com/rambaut/figtree/releases).

### 2.6. Analyses of Single Nucleotide Polymorphisms (SNPs) for Identified Viruses

To identify the mutation positions of identified viruses, we carried out single nucleotide polymorphism (SNP) analyses using only the four libraries derived from single plants. To identify the exact mutation positions, we used assembled virus genome sequences in each library as reference virus genome sequences. For example, assembled viral genomes for SYMMV, PSV RNA1-3, and SYCMV were used as reference genomes for the CHBU-139 library, while a single SMV genome was used as a reference genome for GAWO-62. In the case of GYBU-92, SMV, PSV RNA3, SYCMV, and PeMoV were used for SNP analysis, while three assembled viral genomes—SYMMV, SMV, and SYCMV—were used as the reference genomes for the GYGI library. We carried out SNP analysis as described previously [18]. In brief, we aligned the raw sequence reads on the assembled viral genome using the BWA program with default parameters [22]. The obtained sequence alignment map (SAM) files were converted into binary alignment map (BAM) files using the SAMtools program [29]. Next, the sorted BAM files were converted into the variant call format (VCF) file format using the mpileup function of SAMtools. Finally, SNP calling was conducted using BCFtools implemented in SAMtools. We used the Tablet program to indicate the positions of identified SNPs [30].

### 2.7. RT-PCR Assay

To examine infection of virus in 172 soybean leaf samples, we carried out RT-PCR for five viruses such as SMV, SYMMV, SYCMV, PeMoV, and PSV with newly designed primer pairs. In addition, to confirm the results of RNA-seq, we newly designed RT-PCR primer pairs for three identified viruses: *Tomato spotted wilt virus* (TSWV) in the genus *Tospovirus*, *Bean common mosaic virus* (BCMV) in the genus *Potyvirus*, and *Bean common mosaic necrosis virus* (BCMNV) in the genus *Potyvirus*. The soybean actin gene was used as a positive control, as described previously [31]. The identical total RNAs used for RNA-seq were also used as the template for RT-PCR. We carried out RT-PCR using the DiaStar OneStep RT-PCR Kit (SolGent, Daejeon, Korea). As described previously [17], the following RT-PCR conditions were used: 50 °C for 30 min, 95 °C for 15 min followed by 30 cycles at 95 °C for 20 s, 50 °C to 56 °C for 40 s (the annealing temperature can be variable depending on Tm values of primers), and 72 °C for 1 min, with a final extension at 72 °C for 5 min. The amplified RT-PCR products were checked by gel electrophoresis followed by ethidium bromide staining. All amplified RT-PCR products were cloned in the pGEM-T-Easy Vector (Promega, WI, USA) followed by Sanger sequencing to confirm sequences of amplified PCR products.

## 3. Results

### 3.1. Sample Collection and Examination of Virus Infection by RT-PCR

**Figure 1:**
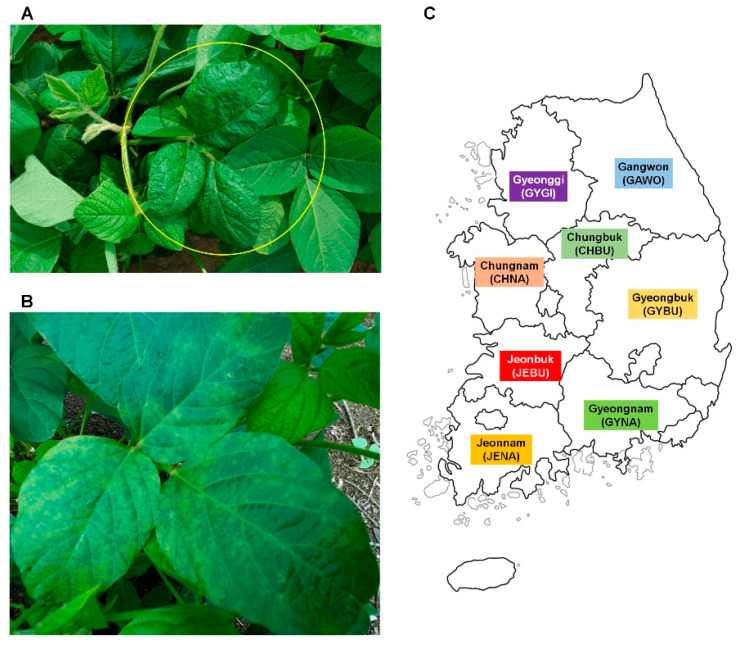
Soybean viral disease symptoms and eight major provinces in the Republic of Korea for soybean virome study. Soybean leaf sample showing viral disease symptoms such as leaf mottling collected from Gyeongbuk (**A**) and Gangwon (**B**). Leaf sample showing viral disease symptoms indicated by yellow-colored circle used for virome study. (**C**) Map displaying eight major provinces in the Republic of Korea from which soybean leaf samples were collected.

We collected 172 soybean leaf samples showing viral disease symptoms from major soybean-growing regions. As shown in Figure 1A,B, not all soybean plants showed viral disease symptoms, and we collected only leaf samples showing viral disease symptoms for the virome study.

We examined infection of virus in 172 soybean leaf samples by RT-PCR using virus-specific primer pairs for five viruses: SMV, SYMMV, SYCMV, PeMoV, and PSV. RT-PCR results showed that most soybean samples except three samples were infected by at least a single virus (Table S1). We grouped samples according to the geographical regions (provinces) (Table 1 and Figure 1C). Out of the examined viruses, three to four were identified from each province. In Gangwon province, SMV (35 samples) was the most frequently identified virus followed by SYMMV (16 samples). Among eight provinces, SMV was the most frequently identified in three provinces (Gangwon, Gyeonggi, and Gyeongbuk). In the other five provinces (Chungbuk, Chungnam, Jeonbuk, Jeonnam, and Gyeonggnam) SYMMV was the most frequently identified virus. SYMMV was identified in all provinces except Gangwon. PeMoV was identified in all provinces except Gyeonggi. PSV was only identified in Gyeonggi. We selected four single soybean samples, which were co-infected by at least three viruses for the NGS-based virome study. SMV and SYMMV were detected in all four single samples, while SYMMV was detected in all single samples except GAWO-62. The proportion of virus infection for SMV ranged from 13 to 100% in the examined soybean samples, whereas that for SYMMV ranged from 46 to 100%.

Next, we examined the number of coinfected viruses in each sample (Table 2). We identified three samples without virus infection by RT-PCR, suggesting that they might be infected by other viruses. Double infection (82 samples) was dominant, followed by single infection (47 samples) and triple infection (38 samples). In addition, two samples, GYBU-92 and GYGU-106, were infected by at least four different viruses.

### 3.2. Library Preparation for Soybean Virome Study

To examine viruses infecting soybean by NGS, the same amount of individual leaf sample was pooled according to the eight provinces in Korea. For example, 35 samples from Gangwon Province were pooled, while 14 samples from Gyeonggi Province were pooled. In addition, we selected four single samples (GAWO-62, GYGI-106, CHBU-139, and GYBU-92), which were coinfected by different viruses to examine the virome in single soybean plants. The four single plants used for NGS were not included in the pooled samples. As a result, eight libraries representing eight major provinces in the Republic of Korea and four libraries from four single soybean plants were generated for RNA sequencing (RNA-seq) (Table 3).

### 3.3. Identification of Viruses Infecting Soybean by RNA-Seq Followed by Bioinformatics Analyses

**Figure 2:**
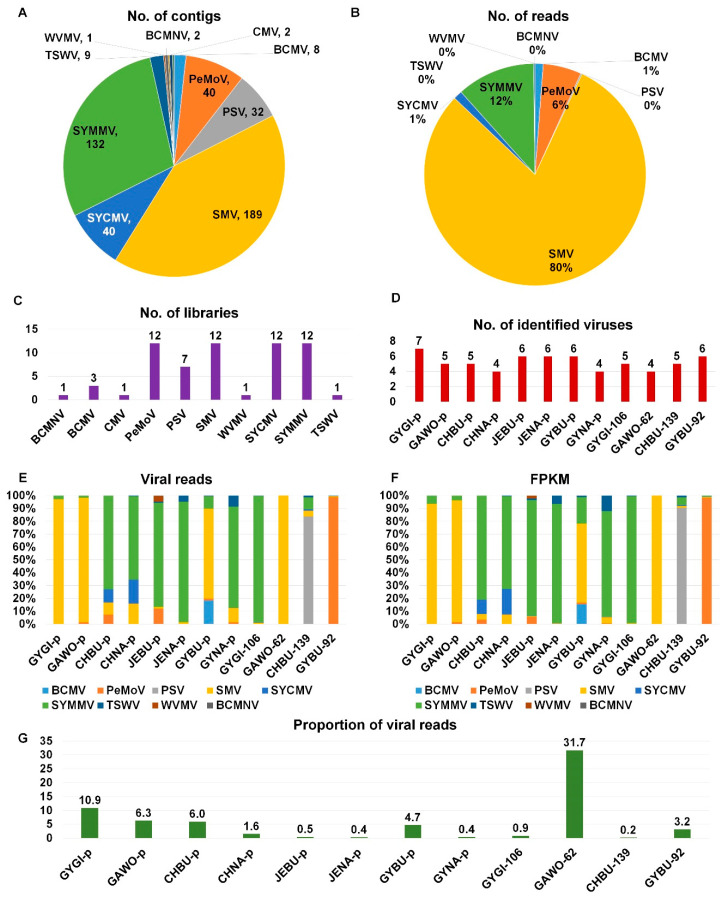
Identification of viruses and viral populations from 12 different libraries. Pie charts displaying the proportion of identified viruses based on the number of contigs (**A**) and number of viral reads (**B**). (**C**) Number of libraries in which individual viruses were identified. (**D**) Number of identified viruses in each library. Proportion of identified viruses based on viral reads (**E**) and fragments per kilobase of transcript per million mapped reads (FPKM) values (**F**) in each library. (**G**) Proportion of viral reads in each library. Abbreviations: Gangwon (GAWO); Gyeonggi (GYGI); Chungbuk (CHBU); Chungnam (CHNA); Jeonbuk (JEBU); Jeonnam (JENA); Gyeongbuk (GYBU); Gyeongnam (GYNA); number (No.); pooled (p).

We conducted RNA-seq, and all raw sequence data were deposited in NCBI’s SRA database with respective accession numbers (Table S2). We identified 455 virus-associated contigs from 12 libraries representing 10 different viruses (Table S3). According to the number of virus-associated contigs, SMV (189 contigs) was the dominant virus, followed by SYMMV (132 contigs), SYCMV (40 contigs), PeMoV (40 contigs), and PSV (32 contigs) (Figure 2A). In addition, we identified TSWV in the family Tospoviridae (nine contigs), BCMV in the family Potyviridae (eight contigs), BCMNV in the family Potyviridae (two contigs), cucumber mosaic virus (CMV) (two contigs) in the family Bromoviridae, and WVMV in the family Potyviridae (one contig). Based on the number of virus-associated reads, SMV (80%) was again the dominant virus, followed by SYMMV (12%) and PeMoV (6%) (Table S4 and Figure 2B). Four viruses (PeMoV, SMV, SYCMV, and SYMMV) were identified in all 12 libraries, while BCMNV, CMV, WVMV, and TSWV were each identified from a single library (Figure 2C). The number of identified viruses in each library ranged from four to seven (Figure 2D). Interestingly, the four individual single plants were also infected by four to six viruses.

### 3.4. Proportion of Identified Viruses in Each Library

Next, we examined the proportion of identified viruses according to virus-associated reads in each library (Figure 2E). Although all libraries were coinfected by different viruses, there was clearly a dominant virus in each library: SMV in four libraries (GYGI-p, GAWO-p, GYBU-p, and GAWO-62), SYMMV in six libraries (CHBU-p, CHNA-p, JEBU-p, JENA-p, GYNA-p, and GYGI-106), PSV in CHBU-139, and PeMoV in GYBU-92 (Figure 2E). We also examined the proportion of identified viruses according to fragments per kilobase of transcript per million mapped reads (FPKM) values (Figure 2F). Only the proportion of SYMMV was slightly increased in the viral proportion based on FPKM values.

In virome studies, it is very difficult to enrich virus particles. In general, most reads sequenced by RNA-seq are derived from plant hosts. We examined the proportion of virus-associated reads in each library (Figure 2G). Unexpectedly, the proportions of virus-associated reads in several libraries, such as GYGI-p (10.9%), GAWO-p (6.3%), CHBU-p (6.0%), and GYBU-p (4.7%), were very high. Surprisingly, the proportion of viral reads in GAWO-62 (31.7%) was the highest in all 12 libraries.

### 3.5. Viral Genome Assembly and Phylogenetic Relationships of Identified Virus Species

By RNA-seq, we could assemble complete or nearly complete viral genome sequences covering whole ORFs: WVMV (one isolate), PeMoV (five isolates), SYMMV (nine isolates), SYCMV (six isolates), PSV RNA1 (two isolates), PSV RNA2 (two isolates), PSV RNA3 (three isolates), and SMV (nine isolates) (Table S5). In addition, we also provide partial viral sequences identified from this study (Table S6).

**Figure 3:**
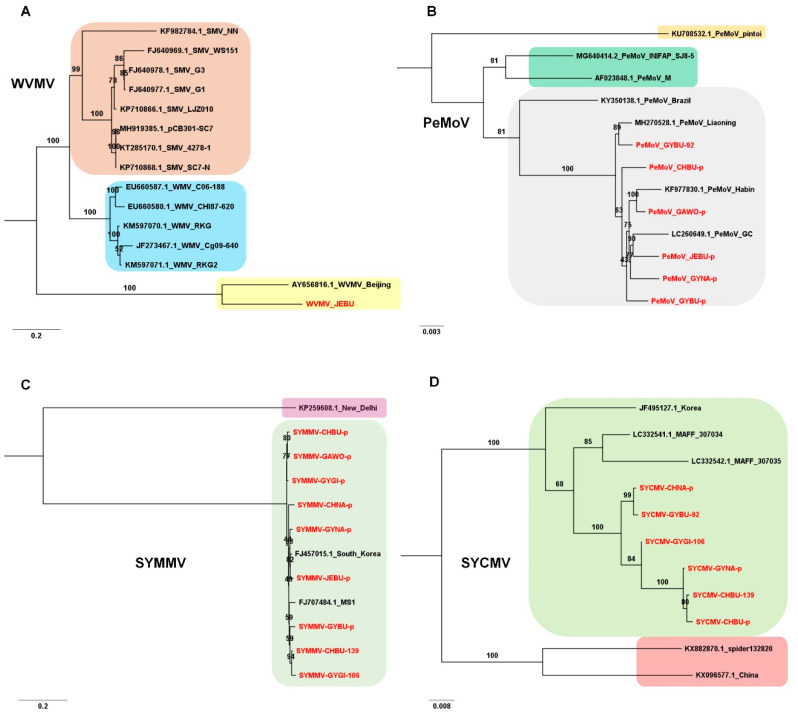
Phylogenetic relationship of WVMV, PeMoV, SYMMV, and SYCMV. Phylogenetic trees of WVMV (**A**), PeMoV (**B**), SYMMV (**C**), and SYCMV (**D**) constructed using complete genome sequences for individual virus species. Nucleotide sequences were aligned by MAFFT, and phylogenetic trees were constructed using IQ-TREE with maximum likelihood method and bootstrap with 1000 iterations. Virus genomes derived from this study are indicated by red color. Best-fit models for each virus species are as follows: GTR+F+I+G4 (WVMV), TN+F+I (PeMoV), TIM2e+I+G4 (SYMMV), and TIM2e+I+G4 (SYCMV). The generated phylogenetic trees were visualized by the FigTree (version 1.4.4).

We obtained the genome sequence of WVMV isolate JEBU, 9668 nucleotides (nt) in length, from Jeonbuk Province by RNA-seq, RT-PCR, cloning, and Sanger sequencing. The phylogenetic tree and BLASTN search demonstrated that WVMV isolate JEBU showed sequence similarity to WVMV isolate Beijing, with 92% coverage and 79.40% nucleotide identity (Figure 3A).

Five complete genomes of PeMoV were obtained from six different libraries. PeMoV isolate GYNA-p covering 98.73% of the complete genome was also included. The sizes of assembled PeMoV ranged from 9603 nt (CHBU-p) to 9708 nt (GYBU-92). Out of 13 available PeMoV genomes, six PeMoV genomes were derived from this study. All six PeMoV isolates in this study were grouped together with other isolates from Korea (two isolates from adzuki bean and soybean, respectively) and China (one isolate from peanut) (Figure 3B).

In the case of SYMMV, we assembled genomes for nine SYMMV isolates whose sizes ranged from 3948 nt (CHBU-p) to 4008 nt (GYGI-106). Except nine SYMMV isolates, there were only three available genome sequences for SYMMV. The SYMMV isolate New Delhi from India was distantly related to other isolates. Out of 11 SYMMV isolates that were grouped together, 10 were identified from Korea, while MS1 was identified from North America (Figure 3C).

We assembled six SYCMV genomes whose sizes ranged from 4077 nt (GYGI-106) to 4152 nt (CHBU-p and CHNA-p). The phylogenetic tree showed that six SYCMV isolates in this study were grouped together with three known SYCMV isolates (two from Japan and one from Korea) (Figure 3D). Two isolates from China, isolate spider132830 from spider and isolate China from soybean, were distantly related to other known SYCMV isolates.

**Figure 4:**
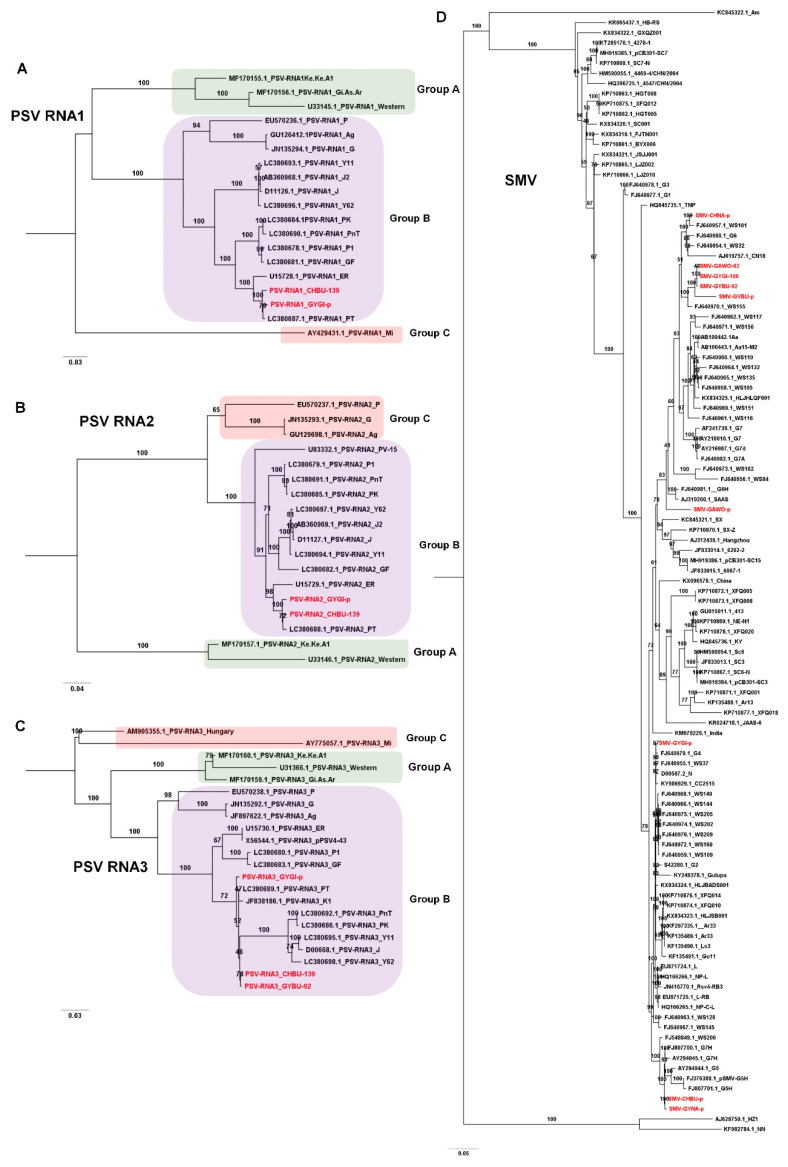
Phylogenetic relationship of PSV RNA1, PSV RNA2, PSV RNA3, and SMV. Phylogenetic trees of PSV RNA1 (**A**), PSV RNA2 (**B**), PSV RNA3 (**C**), and SMV (**D**) constructed using complete genome sequences for individual virus species. Nucleotide sequences were aligned by MAFFT, and phylogenetic trees were constructed using IQ-TREE with maximum likelihood method and bootstrap with 1000 iterations. Virus genomes derived from this study are indicated by red color. Best-fit models for each virus species are as follows: TIM2+F+I+G4 (PSV_RNA1), TN+F+G4 (PSV_RNA2), TIM2+F+G4 (PSV_RNA3), and GTR+F+I+G4 (SMV). The generated phylogenetic trees were visualized by the FigTree (version 1.4.4).

The PSV genome is composed of three RNA fragments. We obtained the three complete RNA fragments for the PSV genome from two libraries, CHBU-139 and GYGI-p. In addition, we obtained the complete PSV RNA3 fragment from the GYBU-92 library. Based on nucleotide sequences for PSV RNA1 (Figure 4A), RNA2 (Figure 4B), and RNA3 (Figure 4C), all PSV isolates from this study belonged to Group B. In particular, the CHBU-139 and GYGI-p were closely related based on the phylogenetic tree.

We assembled nine SMV genomes, and their genome sizes ranged from 9565 nt (GYBU-92) to 9586 nt (CHBU-p). The phylogenetic tree using 111 SMV genomes including nine SMV isolates in this study demonstrated that most SMV genomes were very closely related except for Am (KC845322.1) from Atractylodes macrocephala in China, HZ1 (AJ628750.1) from Pinellia ternate in China, and NN (KF982784.1) from Pinellia pedatisecta in China (Figure 4D). Of the nine SMV isolates, four (GAWO-62, GYGI-106, GYBU-92, and GYBU-p) were in the same clade as the known isolate WS155 from wild soybean in Korea. The isolate CHNA-p was closely related to WS101 from wild soybean in Korea and belonged to the same clade as the G6 strain. The isolate GAWO-p was in the same clade containing the G6H strain, while GYGI-p was closely related to the G4 strain. Two closely related CHBU-p and GYNA-p isolates were in the same clade containing the G7H, G5, and G5H strains.

### 3.6. Analysis of SNPs for Viruses Infecting Soybean in Single Plants

RNA viruses often exist as viral populations composed of an extremely large number of variants referred to as viral quasispecies [32]. In order to examine the composition of viral quasispecies for naturally coinfected RNA viruses, we analyzed SNPs for viruses identified from samples of four single soybean plants named CHBU-139, GAWO-62, GYBU-92, and GYGI-106. We used assembled viral genomes from each library as reference genomes for SNP analysis (Table S7). For example, three assembled viral genomes (PSV, SYCMV, and SYMMV) from the CHBU-139 sample were used as the reference viral genomes for SNP analysis. We identified 23 SNPs (PSV), 0 SNPs (SYCMV), and 189 SNPs (SYMMV) from CHBU-139 (Table 4). From GAWO-92, we only identified a single SNP for SMV. From the GYBU-92 library, we identified 1 SNP (PeMoV), 0 SNPs (PSV), 89 SNPs (SMV), and 0 SNPs (SYCMV). We identified 15 SNPs (SMV) and 0 SNPs (SYCMV) from GYGI-106.

**Figure 5:**
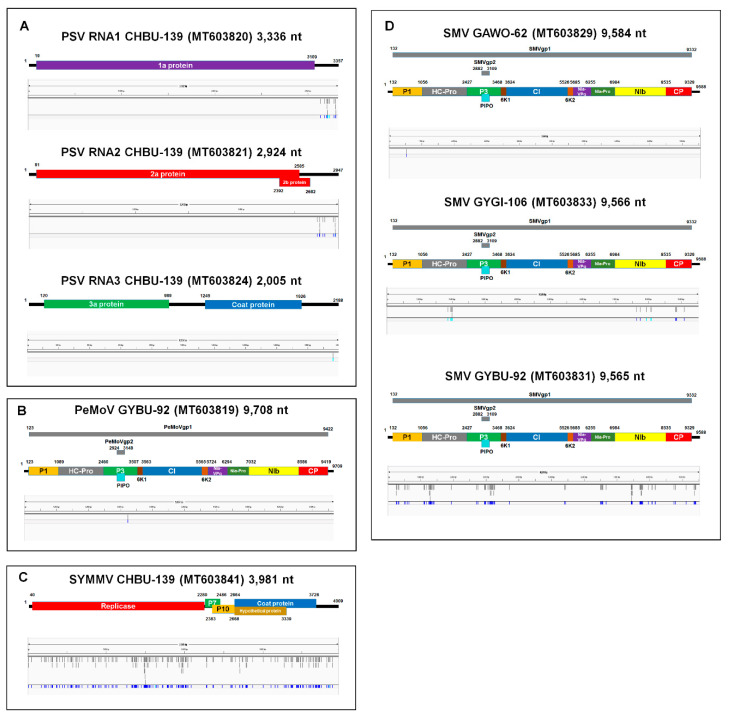
Identification of SNPs for viruses from four single plants. (**A**) Genome structure of PSV and positions of identified SNPs. (**B**) Genome structure of PeMoV and positions of identified SNPs. (**C**) Genome structure of SYMMV and positions of identified SNPs. (**D**) Genome structure of SMV and positions of identified SNPs. We used a reference genome for the genome structure of individual virus species. Information of the complete viral genome sequences used for SNP analysis, including isolate name, accession number, and genome size, is indicated. The SNPs were visualized by the Tablet program.

We examined the positions of identified SNPs (Table S7 and Figure 5). All identified SNPs from PSV RNA fragments from CHBU-139 and GYBU-92 were localized at the 3′ region, and none of them were localized in the ORF region (Figure 5A). From PeMoV isolate GYBU-92, one identified SNP was localized in the P3 protein region (Figure 5B). In the case of SYMMV, there were two isolates (Figure 5C). SYMMV isolate CHBU-139 showed a high number of SNPs distributed on the whole genome of SYMMV, while SYMMV isolate GYGI did not have any SNPs. The single SNP identified from SMV isolate GAWO-62 was localized in the P1 region, while those from SMV isolate GYGI-106 were localized in P1, HC-Pro, CI, Nia-VPg, Nia-Pro, Nib, and CP (Figure 5D). In particular, the number of SNPs for SMV isolate GYGI-106 was higher in the 3′ region than in the 5′ region. In the case of SMV isolate GYBU-92, SNPs were found in the P1, HC = Pro, P3, CI, Nia-Pro, Nib, and CP regions. None of the three SYCMV isolates had SNPs.

Next, we examined the types of identified SNPs (Table 5). In the case of SMV isolate GYBU-92, the number of mutations associated with transitions (Ts) (purine-to-purine or pyrimidine-to-pyrimidine changes), including A→G (25 SNPs), C→T (18 SNPs), G→A (13 SNPs), and T→C (16 SNPs) was high. The number of mutations (17 SNPs) associated with transversions (Tv) (purine–pyrimidine changes) for SMV isolate GYBU-92 was lower than that of transitions (72 SNPs), resulting in a 4.24 Ts/Tv ratio. The number of mutations (9 SNPs) associated with transitions for SMV isolate GYGI-106 was 1.8 times higher than that of transversions (5 SNPs). Similarly, the Ts/Tv ratio for SYMMV isolate CHBU-139 was 7.4 (164 SNPs/22 SNPs). In the case of PSV isolate CHBU-139, the Ts/Tv ratios for the two RNA fragments were 3 (PSV RNA1) and 1.5 (PSV RNA2).

### 3.7. Development of RT-PCR Primer Pairs to Diagnose Eight Major Viruses Infecting Soybean

**Figure 6:**
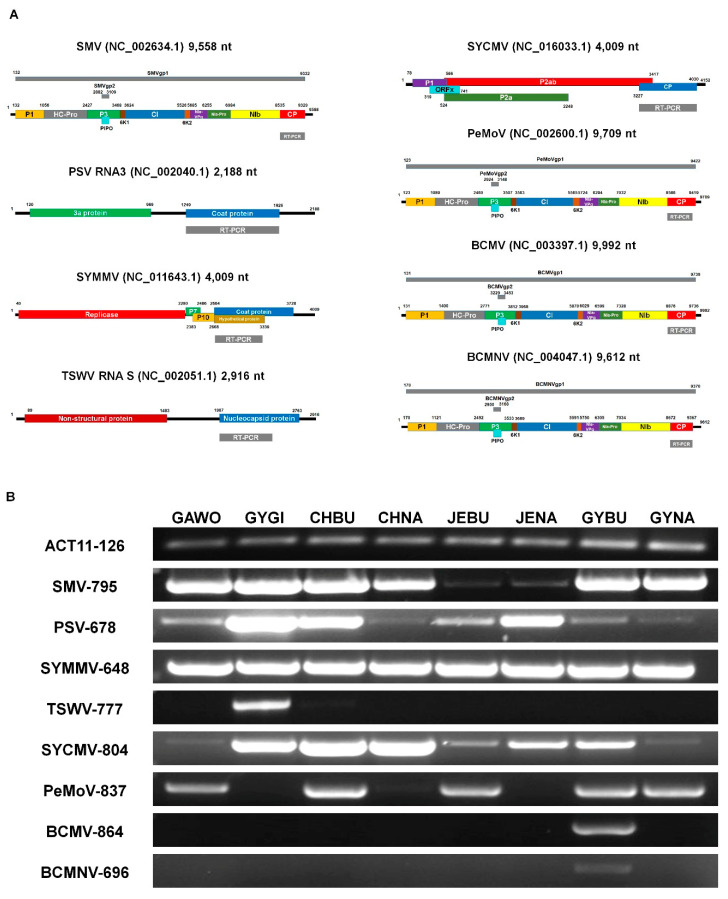
Confirmation of eight major viruses infecting soybean by RT-PCR. (**A**) The individual viral genome structure displays the target virus sequence position amplified by newly designed primer pairs indicated by gray-colored bar. The detailed information of primer pairs can be found in Table S8. (**B**) Amplified PCR products by RT-PCR were visualized by agarose gel electrophoresis. The Actin11 gene of soybean was used as a positive control. We used the same total RNA for both RNA-seq and RT-PCR.

To confirm the RNA-seq results and to develop molecular diagnosis methods for eight major viruses, we newly designed RT-PCR primer pairs based on known reference viral genomes (Table S8). We used sequences encoding coat protein (CP) for SMV, SYMMV, SYCMV, PeMoV, BCMV, and BCMNV (Figure 6A). In the cases of PSV and TSWV composed of three RNA fragments, we used sequences encoding CP and nucleocapsid protein (NC) from PSV RNA3 and TSWV RNA3 fragments, respectively (Figure 6A). The sizes of amplified RT-PCR products ranged from 678 bp (PSV) to 864 bp (BCMV). For RT-PCR, we used the same total RNAs used for RNA-seq. The RT-PCR results using the primer pair for the actin gene of soybean used as a positive control indicated the high quality of extracted total RNAs (Figure 6B). The RT-PCR results were consistent with those of RNA-seq. For example, three major viruses, SMV, SYMMV, and SYCMV, identified from the 12 libraries, were also identified by RT-PCR in all 12 libraries (Figure 6B). TSWV, BCMV, and BCMNV showed region-specific infection by RT-PCR. For example, TSWV was identified only from GYGI, while BCMV and BCMNV were identified from GYBU. RT-PCR confirmed infection of PeMoV in five libraries (except GYGI, CHNA, and JENA).

## 4. Discussion

There have been many studies reporting viruses infecting soybean; however, no large-scale soybean virome study has been conducted. We investigated virus infection for five major viruses in an individual soybean sample by RT-PCR. Moreover, with the help of RNA-seq, we examined viruses infecting soybean in eight provinces in the Republic of Korea by pooling samples according to province and four single soybean plants. Our two different approaches effectively unveiled a wide range of information associated with the soybean virome.

Poly(A) RNA-based enrichment has been used for both RNA and DNA viruses, but it is not applicable for the detection of viruses without a poly(A) tail [33]. However, our several previous virome studies using RNA sequencing with mRNA libraries demonstrated that different types of viral genomes, regardless of the presence of poly(A) tail, can be identified by RNA sequencing with polyA selection [16,17,18]. In addition, currently, preparation of mRNA library is much cheaper than library deleting ribosomal RNAs. Therefore, we used the polyA selection method instead of a method deleting ribosomal RNAs, which was frequently used for virome study. Interestingly, we could recover genomes of two viruses with poly(A) tail, SMV and PeMoV, and three viruses that are not polyadenylated, SYMMV (carmoviruses), SYCMV (sobemoviruses), and PSV (cucumoviruses), using mRNA libraries. There are several possible scenarios for the identification of viruses without poly(A) tail using mRNA library. The first explanation is that a virus, regardless of DNA and RNA genomes, that produces mRNA with poly(A) tail, in any of its life stages, can be detected. The second explanation is that poly(A) selection with oligod(T) is just an enrichment that cannot fully eliminate the other RNAs. Therefore, many remaining viral RNAs can be detected by mRNA sequencing. In general, we could obtain complete viral genomes from the plant samples showing severe viral disease symptoms, suggesting that plant materials are an important factor for virus detection using NGS. To date, the most common approach for virus detection might be to use two different approaches, such as NGS and PCR.

A previous study demonstrated that three viruses (SMV, SYMMV, and SYCMV) were the most important viruses infecting soybean in 2014 by RT-PCR [34]. Consistent with the previous report, we again demonstrated that major viruses infecting soybean in the Republic of Korea were SMV, SYCMV, and SYMMV in 2016 by RT-PCR and RNA-seq. In addition, we revealed that PeMoV and PSV were also frequently identified in the Republic of Korea. Moreover, we identified CMV, TSWV, WVMV, BCMV, and BCMNV infecting soybean in Korea, as previously reported [35,36]. Of them, we report infection of WVMV in the soybean for the first time in Korea and the world. WVMV causes wisteria mosaic disease in wisteria species in the family *Fabaceae* [37]. The genome of WVMV is closely related to SMV and watermelon mosaic virus (WMV) [38]. The obtained complete genome sequence of WVMV isolate JEBU was very different from that from wisteria species [38]. Although several other viruses, including alfalfa mosaic virus (AMV), cowpea mosaic virus (CPMV), soybean dwarf virus (SbDV), and clover yellow vein virus (ClYVV), infecting soybean plants have been identified in Korea [39], we did not find any viral sequences associated with those viruses, suggesting that they are not major viruses infecting soybean in Korea.

Sample pooling is an effective way to monitor virus infection with a large number of samples, and it has many advantages, such as the reduction in experimental costs and analysis time [40]. One of the main purposes of this study was to find major viruses infecting soybean according to different geographical regions (provinces). As we did previously, we again examined virus infection in individual soybean samples by RT-PCR, which was time-consuming and labor-intensive. To compare RT-PCR with NGS, we adopted the sample pooling approach in our study by pooling samples according to eight provinces. Our sample pooling method followed by RNA-seq effectively revealed viral populations in each province. Both RT-PCR and RNA-seq successfully revealed the dominant virus in each province. For example, for the first time, we demonstrated that SMV was the dominant virus in three provinces: Gyeonggi, Gangwon, and Gyeongbuk. SYMMV was the dominant virus in the other five provinces, although there were several viruses infecting soybean in each province. Gyeonggi and Gangwon Provinces are in the northern part of South Korea. Taken together, our soybean virome study revealed that the geographical region plays an important role in the distribution of viruses infecting soybean.

It is known that several potyviruses, including SMV and PeMoV, are transmitted by seeds and aphids, resulting in serious reduction in yield [41,42]. In addition, seed transmission of SYMMV [43] and PSV [44] has been reported. Of the five major viruses infecting soybean in Korea, seed transmission has been examined in all but SYCMV. We tentatively assume that the seed transmission ability of major soybean viruses might be one of the major factors of the incidences of several viruses infecting soybean in Korea. In general, the same soybean cultivars in each region are planted for a long time. Moreover, most soybean growers in Korea use seeds derived from the harvest of the previous year. Therefore, the soybean seeds produced by farmers might be reservoirs of viruses infecting soybean in Korea. As a result, the production and supply of virus-free soybean seeds are recommended to reduce the virus incidence of soybean in Korea, as suggested previously [34]. However, the possibility of virus transmission in soybean plants by insects is also high in Korea.

According to RT-PCR, all four single plants were coinfected by three to four viruses. By contrast, RNA-seq identified four to six viruses from the same sample. For example, except GAWO-62, all single plants were coinfected by two potyviruses (SMV and PeMoV), PSV, SYCMV, and SYMMV. Similarly, NGS identified four to seven viruses from pooled samples. As compared to RT-PCR diagnosing only known viruses, RNA-seq revealed additional unidentified viruses. This result showed the advantage of NGS over RT-PCR in virus identification. We found that coinfection of several viruses in soybean plants was common in Korea, as previously reported [34,45]. The approach using RT-PCR to reveal the distribution of coinfection in soybean samples was successful, revealing that coinfections, such as double and triple infections, were common in soybean plants grown in the Republic of Korea. Moreover, in many cases, double and triple infections in soybean in the Republic of Korea were caused by three major viruses: SMV, SYMMV, and SYCMV. This result suggests that these three viruses should be properly controlled in the Republic of Korea to prevent the spread of virus diseases in soybean.

Although the RT-PCR method revealed the number of viruses infecting individual soybean samples, we could not identify the dominant virus in each sample. With the help of RNA-seq with four different single samples, we found that there was no common dominant virus in the four single samples. In each single sample coinfected by different viruses, the dominant virus varied. This result indicates that several factors, including the virulence of infecting viruses, environmental conditions, and plant hosts, might affect the viral disease symptoms in coinfected soybean.

In this study, we revealed the list of coinfected viruses in each sample; however, we did not know whether those viruses were simultaneously (coinfection) or sequentially (superinfection) infected [46]. In natural conditions, triple, quadruple, or higher coinfection of different viruses might be achieved by sequential infection. The most interesting result of coinfected viruses in soybean was that one major virus in the single plants was dominantly accumulated (e.g., SYMMV in GYGI-106 and SMV in GAWO-62), and it varied depending on the plant sample.

The proportion of virus-associated reads by RNA-seq is generally very low. Surprisingly, the proportions of virus-associated reads for four pooled samples—GYGI-p, GAWO-p, CHBU-p, and GYBU-p—and a single plant, GAWO-62, were higher than 4%. Except CHBU-p, SMV was dominantly present in the four libraries. In particular, the 31.7% in the GAWO-62 sample was the highest viral proportion value in diverse plants infected by viruses and viroids.

With the help of RNA-seq, we assembled several viral genomes that can be usefully applied for diverse analyses, such as phylogenetic and mutation analyses. We found that different hosts and different geographical regions contribute to the genetic diversity of RNA viruses. For example, phylogenetic analysis showed that the genomes of SYMMV and PeMoV derived from Korea are highly conserved; however, SYMMV isolate from New Delhi in India, two PeMoV isolates from wild Phaseolus species in Mexico, and one isolate from *Arachis pintoi* in Brazil were distantly related to those from Korea. Similarly, two SYCMV isolates from China were distantly related to SYCMV isolates from Korea. Furthermore, the genomes of three SMV isolates (Am, HZ1, and NN) in China were different from those of other SMV isolates from soybean, since the three SMV isolates were not identified from soybean. These results strongly suggest that the host plays an important role in the genetic diversity of RNA viruses.

SNP analysis revealed the quasispecies of identified RNA viruses. As previously reported, the frequency of transition was higher than that of transversion [47]. Interestingly, there was no mutation for three SYCMV isolates, indicating a single SYCMV variant infection in the three single plants. Both PeMoV and SMV are potyviruses; however, the number of identified SNPs for SMV was much higher than that of PeMoV. The numbers of identified SNPs among the three SMV isolates differed significantly from each other, ranging from 1 (GAWO-62) to 89 (GYBU-92), suggesting that the mutation rate of SMV could be changeable depending on the soybean plant and the combination of coinfected viruses. Although we obtained three RNA fragments of PSV from CHBU-139 and GYBU-92, the number of identified SNPs was much higher in CHBU-139 (23 SNPs) as compared to GYBU-92 (0 SNPs). It is noteworthy that the level of PSV was much higher in CHBU-139 than in GYBU-92, indicating that the level of viral accumulation for PSV might be related to the mutation frequency. The number of SNPs for SYMMV isolate CHBU-139 was very high, while SYMMV isolate GYGI-106 did not have any SNPs, indicating that the quasispecies of SYMMV were also dependent on the soybean plant and the combination of coinfected viruses. The high level of virus accumulation was not always correlated to the mutation frequency. For instance, SMV was the dominant virus in the GAWO-62 plant, which was coinfected by at least five viruses; however, a single SNP was identified. In addition, coinfection of different viruses did not enhance the mutation rate for a certain virus. Taken together, the results suggest that not a single factor but numerous unknown factors (including hosts, viruses, and environmental conditions) might regulate the accumulation and mutation of coinfected viruses.

## 5. Conclusions

Our soybean virome study using RNA-seq identified a total of 10 different viruses infecting soybean plants. Our comprehensive analysis revealed viral populations in eight different provinces in the Republic of Korea and four different single plants in detail. Out of the 10 identified viruses, five were major viruses infecting soybean in Korea, and there was a single dominant virus in each province, although we identified several viruses infecting soybean. The geographical difference in the dominant virus infecting soybean in each province might be related to the soybean seeds that transmit viruses. In this study, 32 viral genome sequences were assembled and successfully used to analyze the phylogenetic relationships and quasispecies nature of the identified RNA viruses. Coinfection of different viruses in soybean plants was very common in Korea and resulted in severe viral disease symptoms. The viral population as well as virus accumulation and mutation rate of individual viruses in single plants with coinfection of different viruses varied depending on several unknown factors, including hosts, viruses, and environmental conditions.

## Figures and Tables

**Table 1:** Summary of virus infection for 172 soybean leaf samples by RT-PCR.

Group	SMV	SYMMV	SYCMV	PeMoV	PSV	No. of Samples	No. of Infected Viruses
GAWO-p	35 (100%)	16 (46%)	0 (0%)	5 (14%)	0 (0%)	35	3
GYGI-p	14 (100%)	9 (64%)	3 (21%)	0 (0%)	1 (7%)	14	4
CHBU-p	5 (31%)	15 (94%)	14 (88%)	3 (19%)	0 (0%)	16	4
CHNA-p	9 (60%)	15 (100%)	15 (100%)	1 (7%)	0 (0%)	15	4
JEBU-p	2 (13%)	15 (100%)	6 (40%)	1 (7%)	0 (0%)	15	4
JENA-p	8 (57%)	14 (100%)	7 (50%)	1 (7%)	0 (0%)	14	4
GYBU-p	27 (87%)	23 (74%)	6 (19%)	3 (10%)	0 (0%)	31	4
GYNA-p	9 (32%)	23 (82%)	13 (46%)	1 (4%)	0 (0%)	28	4
GAWO-62	1 (100%)	1 (100%)	0 (0%)	1 (100%)	0 (0%)	1	3
GYGI-106	1 (100%)	1 (100%)	1 (100%)	0 (0%)	0 (0%)	1	3
CHBU-139	1 (100%)	1 (100%)	1 (100%)	0 (0%)	1 (100%)	1	4
GYBU-92	1 (100%)	1 (100%)	1 (100%)	1 (100%)	0 (0%)	1	4
Total	113 (66%)	134 (78%)	67 (39%)	17 (10%)	2 (1%)	172	5

Virus infection for 172 soybean leaf samples was examined by RT-PCR using five virus-specific primer pairs. The numbers and percentages indicate the number and proportion of virus-infected samples, respectively, according to the eight geographical regions and four different single samples. Detailed information on the RT-PCR results can be found in Table S1. P indicates pooled samples according to eight different geographical regions. Four samples (GAWO-62, CHBU-139, GYBU-92, and GYGU-106) were derived from a single plant that was further used for a virome study using next-generation sequencing (NGS). Abbreviations: Gangwon (GAWO); Gyeonggi (GYGI); Chungbuk (CHBU); Chungnam (CHNA); Jeonbuk (JEBU); Jeonnam (JENA); Gyeongbuk (GYBU); Gyeongnam (GYNA); number (No.); pooled (p).

**Table 2:** Number of samples with virus coinfection in eight different provinces and four single samples.

Group	None	Single	Double	Triple	Quadruple
GAWO-p	0	14	21	0	0
GYGI-p	0	4	7	3	0
CHBU-p	1	1	6	8	0
CHNA-p	0	0	5	10	0
JEBU-p	0	7	7	1	0
JENA-p	0	4	4	6	0
GYBU-p	2	6	16	7	0
GYNA-p	0	11	16	1	0
GAWO-62	0	0	0	1	0
CHBU-139	0	0	0	1	0
GYBU-92	0	0	0	0	1
GYGI-106	0	0	0	0	1
Total	3	47	82	38	2

**Table 3:** Summary of 12 libraries used for RNA-seq to identify viruses infecting soybean in Korea. Eight libraries were derived from pooled samples, while four libraries were derived from four single plants.

Library Name	Collected Province	Single or Pooled	No. of Samples
GAWO-p	Gangwon	Pooled	35
GYGI-p	Gyeonggi	Pooled	14
CHBU-p	Chungbuk	Pooled	16
CHNA-p	Chungnam	Pooled	15
JEBU-p	Jeonbuk	Pooled	15
JENA-p	Jeonnam	Pooled	14
GYBU-p	Gyeongbuk	Pooled	31
GYNA-p	Gyeongnam	Pooled	28
GAWO-62	Gangwon	Single	1
GYGI-106	Gyeonggi	Single	1
CHBU-139	Chungbuk	Single	1
GYBU-92	Gyeongbuk	Single	1

**Table 4:** Information of identified number of single nucleotide polymorphisms (SNPs) for viruses from four single plants.

Virus Genome	CHBU-139	GAWO-62	GYBU-92	GYGI-106
PeMoV			1	
PSV-RNA1	12		None	
PSV-RNA2	10		None	
PSV-RNA3	1		None	
SMV		1	89	15
SYCMV	None		None	None
SYMMV	189			None

**Table 5:** Number of identified SNPs according to mutation type. Gray color indicates transition (Ts) mutation type, while other colors indicate transversion (Tv) mutation type.

	PeMoV	SMV			SYMMV	PSV		
SNP	GYBU-92	GAWO-62	GYBU-92	GYGI-106	CHBU-139	RNA1-CHBU-139	RNA2-CHBU-139	RNA3-CHBU-139
A→C			5		3			
A→G	1		25	5	37	2		
A→T			4	2	3	1	2	
C→A		1	2		6	1	1	
C→G			1		2			
C→T			18	2	54	2	1	1
G→A			13	1	26	3	3	
G→T			1	1	2			
G→C			1					
T→A			1	2	5	1	1	
T→C	1		16	1	47	2	2	
T→G			2		1			
A→C,T					1			
T→C,A					1			
TTCT→TT				1				
GTTTT→GTTT					1			

## Supplementary Materials

The following are available online at https://www.mdpi.com/2076-2607/8/11/1777/s1, Table S1. Information of individual soybean samples and RT-PCR results using five virus-specific primers. Table S2. Information of raw sequence data from 12 libraries. All raw sequence data were deposited in NCBI’s SRA database with respective accession numbers. Table S3. Number of virus-associated contigs in 12 libraries. For PSV and CMV composed of three RNA fragments, the number of viral contigs assigned to individual RNA fragments was indicated. Table S4. Number of viral reads, coverage, and FPKM values. Raw sequence reads were mapped on the identified virus reference genome using BWA. The mapped reads were subjected to pileup.sh implemented in the BBMap program to calculate the coverage, number of viral reads, RPKM, number of fragments, and FPKM values. Table S5. List of virus genomes assembled from this study. Information of the assembled virus genomes, including accession number, isolate name, and genome size, is listed. Table S6. Assembled complete and partial genome sequences of identified viruses from this study. Viral genome sequences covering whole ORFs were deposited in NCBI’s GenBank. The predicted protein sequences were also provided. Table S7. Results of SNP analysis. For SNP analysis, assembled viral genomes from each library were used as reference genomes. Libraries from four single plants were used for SNP analysis. Results of SNP analysis for viruses from CHBU-139 sample. Table S8. Primer information for RT-PCR. To confirm the RNA-seq results, we newly designed primer pairs for RT-PCR to diagnose eight major viruses infecting soybean plants. Primers were designed based on viral CP sequences. *Actin* gene from *Glycine max* L. was used as a positive control for RT-PCR.
